# Determining the burden of falls amongst community-dwelling older people in Ireland to inform falls care delivery: secondary data analysis from the Irish longitudinal study on ageing – the defined study

**DOI:** 10.1136/bmjopen-2025-107647

**Published:** 2026-01-30

**Authors:** Robert Briggs, Mark Ward, Siobhan Scarlett, Nathalie van der Velde, Belinda Hernandez, Roman Romero-Ortuno, Bryan Tysinger, Peter May, Emer Ahern, Rose Anne Kenny

**Affiliations:** 1 The Irish Longitudinal Study on Ageing, Trinity College Dublin, Dublin, Leinster, Ireland; 2 Geriatric Medicine, University of Amsterdam, Amsterdam, The Netherlands; 3 Trinity College, Dublin, Ireland; 4 Leonard D. Schaeffer Center for Health Policy and Economics, University of Southern California, Los Angeles, California, USA; 5 King’s College London, London, UK; 6 Geriatric Medicine, Cork University Hospital, Cork, County Cork, Ireland

**Keywords:** Aged, GERIATRIC MEDICINE, Frailty

## Abstract

**Abstract:**

**Objective:**

Falls represent the most frequent reason older people are admitted to hospital and significantly increase the likelihood of functional decline, healthcare utilisation and early mortality. The aim of this study is to comprehensively delineate the burden of falls amongst community-dwelling older people in Ireland.

**Design:**

Population-representative analysis of Wave 6 of the Irish Longitudinal Study on Ageing (TILDA) estimating incidence of falls requiring medical attention and emergency department (ED) attendance, fractures and fear of falling over 12 months. Additional data detailing falls-risk increasing drugs (FRIDs) and prior falls were also analysed.

Using Central Statistics Office Census 2022, the population of older people in Ireland was multiplied by the proportion of TILDA participants with each outcome of interest to yield population-level estimates.

**Participants/Setting:**

Population-representative sample of 2299 (55% female) community-dwelling people in Ireland aged ≥70 years.

**Results:**

Almost 12% (proportion 0.12 (95% CI 0.10 to 0.13)) of participants, corresponding to almost 62 000 older people in Ireland, reported a fall requiring medical attention in 12 months, with 6% (proportion 0.06 (95% CI 0.05 to 0.07)), or over 32 000 people, attending ED due to a fall. Over 3% (proportion 0.03 (95% CI 0.03 to 0.04)) reported sustaining a fracture. Almost half of participants reporting a fall requiring medical attention were prescribed FRIDs, and over half had also reported a fall when assessed at the prior wave of the study (ie, 2 years ago).

**Conclusions:**

The burden of falls amongst community-dwelling older people is considerable; 1 in 8 required medical attention for a fall and 1 in 16 attended the ED with falls over 12 months.

Currently, there is no national falls strategy in Ireland. These findings, alongside our ageing population, underscore the need for strengthened falls-prevention strategies to reduce avoidable morbidity and healthcare utilisation.

**STRENGTHS AND LIMITATIONS OF THIS STUDY**

- Detailed, population-representative analysis of falls in 2299 community-dwelling older people with longitudinal follow-up.
- Captures proportion of falls requiring medical attention and emergency department attendance at a population level amongst older people in Ireland, using census data to estimate true burden of falls.
- Detailed information on use of falls risk-increasing drugs, cross-checked with individual medication lists.
- Data limited to the Irish population, which may impact on the generalisability of findings; however, falls care is similarly underdeveloped across the UK and Europe.
- Falls are elicited by self-report and may be subject to some recall bias.

## Introduction

 The Global Burden of Disease Study estimates that falls result in almost 17 million years of life lost and over 19 million years lived with disability worldwide.^1^ In Europe, death and disability due to falls have been increasing for decades, and given increased longevity, are likely to continue to do so.^2^

At an individual level, falls can have a profound effect on well-being, quality of life and functional independence.^3^ They represent the most common cause of accidental death of older people^4^ and the most frequent reason for presentation to hospital.^5^ Falls, particularly falls causing injury, significantly increase the likelihood of nursing home admission,^6^ prolonged hospital length of stay,^7^ cognitive decline,^8^ fear of falling^9^ and early mortality.^10^

Many falls in later life are potentially preventable, however.^11^ Falls prevention requires careful assessment, with access to multidisciplinary care for those who require it.^12 13^ Multimodal interventions, which may incorporate strategies including strength and balance exercise,^14^ home hazard reduction,^11^ treatment of vision problems^11^ and management of cardiovascular cases of falls,^15^ have been shown to significantly reduce falls risk. A further important component of falls prevention involves deprescribing medications that increase the risk of falls, where feasible.^16^ It is likely that falls-risk increasing drugs (FRIDs) increase falls through several mechanisms, including sedation, disruption of gait and balance and by causing orthostatic hypotension.^17 18^ STOPPFALL (Screening Tool of Older Persons Prescriptions in older adults with high fall risk) has been developed to identify and guide deprescribing FRIDs to support a comprehensive falls prevention assessment.^19^

Age-attuned multidisciplinary care for older people at high risk prevents further falls and the adverse health outcomes related to falls, as well as relieving acute hospital pressures in terms of emergency department (ED) congestion and acute hospital bed capacity.^20^,^22^ Despite this, currently, there is no dedicated falls prevention strategy or programme for falls care in Ireland.

Sláintecare is a government-led 10-year plan currently underway to transform publicly-funded health and social care services in Ireland.^23^ One of the key intentions of Sláintecare is to establish regional health areas to ensure geographical alignment of healthcare services at a local level, based on defined populations and their needs.^24^ This aims to introduce a population-based approach to service planning and ensure healthcare is planned and funded around the needs of the population.^24^

This significant reconfiguration of healthcare in Ireland represents an important opportunity to inform better service planning for patients with falls, and the aim of this study, therefore, is to comprehensively delineate the burden of falls requiring medical care among older people in Ireland. Using population-representative data from the Irish Longitudinal Study on Ageing, we will determine the current demand for a ‘comprehensive multifactorial falls risk assessment and … personalized multidomain interventions’ for older people with falls, as recommended by World Falls Guidelines, as well as the use of FRIDs among this cohort.^13^ We will further assess if the demand for multidisciplinary fall care is being met by mapping falls against current availability of dedicated falls clinics, including across proposed new regional health areas.

Our hypothesis is that the burden of falls among older people is considerable and that a significant proportion of older people with falls cannot access the care they require within their regional health area, and our intention is that the findings from this study can inform future service planning for falls care in Ireland.

## Methods

This study examines the burden of falls among older people in Ireland in the 12 months of 2022. Data from the Irish Longitudinal Study on Ageing (TILDA) is used to estimate the prevalence of falls requiring medical attention, ED attendance due to falls, fractures and fear of falling. Further, we outline the proportion of FRID use and prior falls reported by older people who require medical attention, including ED attendance, after a fall in Ireland.

### Study design

The TILDA study design has been outlined previously.^25^ Briefly, there are three components to data collection: a computer-assisted personal interview carried out by social interviewers in the participants’ own home; a self-completion questionnaire completed and returned by the participant; and a comprehensive centre-based health assessment or a modified home-based health assessment carried out by trained research nurses. Study waves were conducted at 2-year intervals. We primarily analysed data from the sixth wave of TILDA conducted from 2020 to 2022, as well as falls prevalence data from Wave 5, conducted from 2017 to 2018. Participants were included in the analysis of this study if they were aged ≥70 years at Wave 6.

TILDA recruitment at Wave 1 was conducted using a stratified clustered procedure to randomly sample postal addresses from the Irish Geo-Directory (a listing of all residential addresses in Ireland). All postal addresses in Ireland were assigned to one of 3155 geographic clusters; using RANSAM (a random sampling design for Ireland), a sample of 640 of these clusters was selected stratified by socioeconomic group and geography, where all household residents aged ≥50 years were eligible to participate.^26^ Each older person in Ireland had a 1 in 156 chance of being represented in the study. This sampling method ensures that the TILDA cohort is a population-representative sample and that Geocodes are available for each participant.^26^

TILDA geocodes were mapped to newly established regional health areas so that the proportion of community-dwelling older adults requiring falls care within each of the regional health areas could be estimated. These new regional health areas are Area A: Dublin North, Meath, Louth, Monaghan and Cavan; Area B: Longford, Westmeath, Offaly, Laois, Kildare and parts of Dublin and Wicklow; Area C: Tipperary South, Waterford, Kilkenny, Carlow, Wexford, Wicklow and part of South Dublin; Area D: Kerry and Cork; Area E: Limerick, Tipperary and Clare; Area F: Donegal, Sligo, Leitrim, Roscommon, Mayo and Galway.

### Falls

At Wave 6, all TILDA participants were asked ‘Have you had any falls in the last year?’ If they answered affirmatively, they were further asked ‘Were the fall(s) serious enough to require medical attention?’. This informed our ‘Falls requiring medical attention within the last 12 months’ variable.

Participants were then asked if they attended an ED due to a fall. This informed the ‘ED Attendance with Falls’ variable.

Participants were asked to report if they had fractures in the past year. If they answered affirmatively, participants were asked if they had a fractured hip, wrist or vertebra. Prior studies have shown that self-report of fracture history is sufficiently reliable.^27^ As fracture rates were relatively low, data on hip, wrist and vertebral fractures were combined into a single ‘Fractures’ variable.

Participants were also asked ‘Are you afraid of falling?’ If they answered affirmatively, they were then asked ‘Do you feel somewhat afraid or very afraid of falling?’ and ‘Do you ever limit activities because you are afraid of falling?’ Analysis was conducted on those who reported that they were ‘very afraid’ of falling.

Similar data pertaining to falls was collected at Wave 5, and this was also incorporated into the analysis.

### Falls-risk increasing drugs

STOPPFall collates 14 classes of medications that increase the risk of falls in later life.^19^ At Wave 6, medication lists provided by participants were examined for FRIDs defined by STOPPFall, using the Anatomical Therapeutic Chemical (ATC) Classification system. The STOPPFall medication classes comprise benzodiazepines (ATC codes of N03AE, N05BA and N05CD), antipsychotics (ATC code of N05A), benzodiazepine-related drugs (N05CF), opioids (N02A, R05DA), antidepressants (N06A, N06CA), antiepileptics (N03), diuretics (C02L, C03, C07B, C07C, C07D, C08GA, C09BA, C09BX01, C09BX03, C10BX13, C09DA, C09DX01, C09DX03, C09DX06, C09DX07, C09XA52, C09XA54), alpha-blockers used as antihypertensives (C02CA), alpha blockers used for prostate hyperplasia (G04CA), centrally-acting antihypertensives (C02A), antihistamines (N07CA02, R06), vasodilators used in cardiac diseases (C01D) and drugs for urinary frequency and incontinence (G04BD, G04CA53). Anticholinergic medications were defined by a comprehensive list of medications with ‘definite’ anticholinergic effects based on the Anticholinergic Cognitive Burden scale.^28^ Only anticholinergic medication not already classified in other STOPPFall medication classes was included to avoid duplication and included gastrological agents (A03AA07, A03AB05, A03BA01, A03BA03 and A03BB01), Parkinsonian agents (N04AA01, N04AA02, N04AA04, N04AB02 and N04AC01), antihistamines (N02BE51) and other medications (M03BA03, M03BC01, N05CM05 and N05BB01).

### Additional data sources

The Central Statistics Office (CSO) is Ireland’s national statistical institute, providing independent statistics about society, economy and environment, which are freely available to everyone and support evidence-informed decision making.^29^ Data from the CSO Census 2022 were used to extrapolate representative TILDA data to census population numbers.^30^ Data obtained from the CSO comprised the numbers of older people of both sexes aged 70–74 years, 75–79 years, 80–84 years and ≥85 years across each of the six newly established regional health areas.

In total, there were 538 171 people aged ≥70 years living in Ireland in 2022, 202 884 aged 70–74 years, 154 260 aged 75–79 years, 96 586 aged 80–84 years and 84 441 aged ≥85 years. The breakdown of this group by sex and into Regional Health Areas (RHAs) are shown in online supplemental table 1.

Currently, there is no pathway in place within Ireland’s primary care system for older people at risk of falls to have a multifactorial falls risk assessment, and generally patients are referred on to clinics, for example, general medical clinics, geriatric medicine clinics, dedicated falls clinics, for the assessment. Data on access to dedicated falls clinics, that is, dedicated clinics that provide multifactorial risk assessment for older people with falls via a multidisciplinary team, available at each model 3 and model 4 hospital in Ireland were collected.^31^ Data on clinics are publicly available and listed on the respective hospital referral forms/listings and were confirmed locally.

The following acute hospitals were included: Beaumont Hospital Dublin, Cavan General Hospital, Connolly Hospital Dublin, Cork University Hospital, Kerry University Hospital, Letterkenny University Hospital, Mater Misericordiae Hospital Dublin, Mayo University Hospital, Mercy University Hospital Cork, Midland Regional Hospital Mullingar, Midland Regional Hospital Portlaoise, Midland Regional Hospital Tullamore, Naas General Hospital, Our Lady of Lourdes Hospital Drogheda, Our Lady’s Hospital Navan, Portiuncula University Hospital Ballinasloe, Sligo University Hospital, South Tipperary General Hospital, St James’s Hospital Dublin, St Luke’s Hospital Kilkenny, St Vincent’s University Hospital Dublin, Tallaght University Hospital Dublin, University Hospital Galway, University Hospital Limerick, University Hospital Waterford and Wexford General Hospital.

While all older people who fall may not require a multifactorial falls risk assessment, world fall guidelines recommend that any older person who presents to healthcare with a fall causing an injury, ≥2 falls in the last year, frailty, inability to get up from the floor after a fall and/or suspected syncope should have a multifactorial risk assessment.^13^

### Statistical analysis

Data are presented descriptively with proportions and 95% CIs for binary variables.

To allow for attrition since Wave 1, data were weighted against the CSO 2022 Census to ensure that for the estimates presented, subgroups within the sample are represented proportionate to the number of that subgroup present in the population of Ireland. The weights reflect the reciprocal of the probability of a participant being included in the study, based on individual characteristics. This probability was calculated using a multivariate logistic regression model, with baseline predictors including age, sex, educational attainment, marital status, urban or rural residence, self-rated physical health and smoking.

For extrapolation of TILDA data to population-based estimates, the population of males and females in each RHA was multiplied by the proportion of TILDA participants with each outcome of interest (falls requiring medical attention, ED attendance due to falls, fractures and fear of falling).

## Results

2299 TILDA participants were included in the study. Almost 55% (1258/2299) were female. Mean age was 77.43 (SE 0.12) years and the breakdown by age groups 70–74, 75–79, 80–84 and ≥85 years was 39%, 29%, 14% and 14%, respectively.

Almost 40% (917/2299) were prescribed at least one FRID, while 13% (301/2299) were prescribed at least two FRIDs.

Almost one-third of participants (proportion 0.32 (95% CI 0.30 to 0.34)) reported a fall within the last 12 months.

### Falls requiring medical attention

Almost 12% (proportion 0.12 (95% CI 0.10 to 0.13)) of participants reported a fall that required medical attention within the last 12 months, over 10% of males (proportion 0.10 (95% CI 0.08 to 0.13)) and 13% of females (proportion 0.13 (95% CI 0.11 to 0.15)).

**Figure 1:**
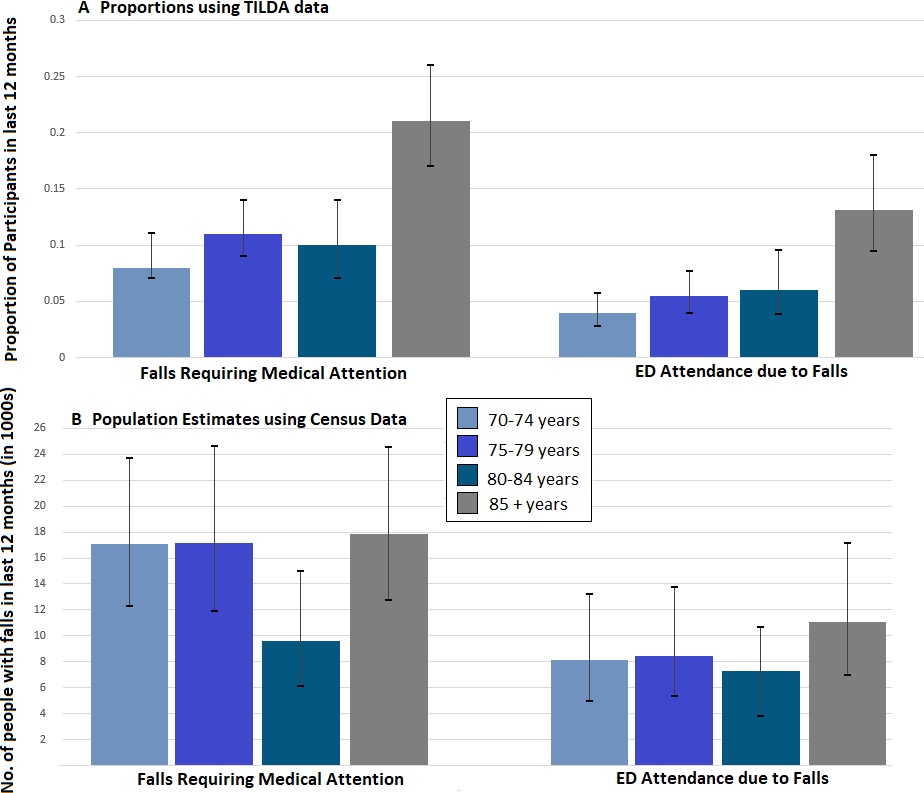
Proportions and population-based estimates of older people with falls in 2022. ED, emergency department; No., number; TILDA, The Irish Longitudinal Study on Ageing; Proportions reported with 95% CIs. Population estimates by extrapolating TILDA data to 2022 census.

Figure 1A demonstrates the breakdown of falls requiring medical attention by age group, with over one-fifth of those aged ≥85 years reporting a fall requiring medical attention within the last 12 months.

**Figure 2:**
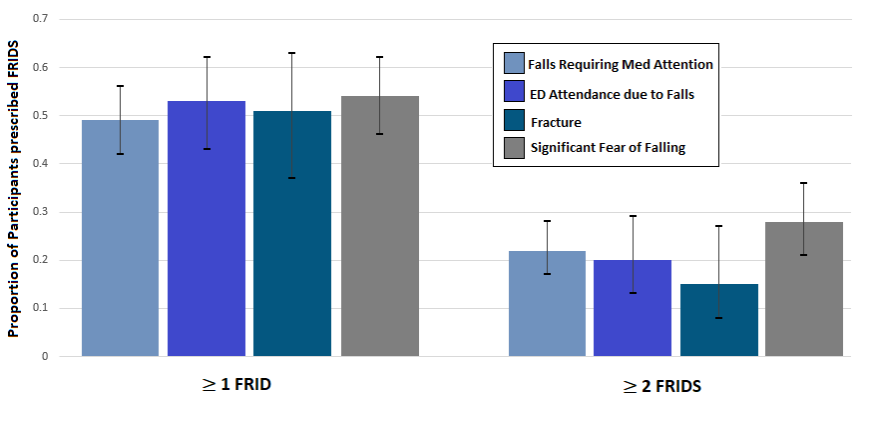
FRID use among older people with falls and fear of falling. ED, emergency Department; FRIDs, falls-risk increasing drugs; Med, medical. Proportions presented with 95% CIs. FRIDs defined using the Screening Tool of Older Persons Prescriptions in older adults with high fall risk (STOPPFALL) and comprise benzodiazepines (ATC codes of N03AE, N05BA and N05CD), antipsychotics (ATC code of N05A), benzodiazepine-related drugs (N05CF), opioids (N02A, R05DA), antidepressants (N06A, N06CA), antiepileptics (N03), diuretics (C02L, C03, C07B, C07C, C07D, C08GA, C09BA, C09BX01, C09BX03, C10BX13, C09DA, C09DX01, C09DX03, C09DX06, C09DX07, C09XA52, C09XA54), alpha-blockers used as antihypertensives (C02CA), alpha blockers used for prostate hyperplasia (G04CA), centrally-acting antihypertensives (C02A), antihistamines (N07CA02, R06), vasodilators used in cardiac diseases (C01D) and drugs for urinary frequency and incontinence (G04BD, G04CA53) and anticholinergic medications with ‘definite’ anticholinergic effects based on the Anticholinergic Cognitive Burden (ACB) scale.

As shown in figure 2, almost half (proportion 0.49 (95% CI 0.42 to 0.56)) of participants reporting a fall requiring medical attention were prescribed a FRID, while over one-fifth (proportion 0.22 (95% CI 0.17 to 0.28)) were prescribed at least two FRIDs.

Over half of participants (proportion 0.52 (95% CI 0.45 to 0.59)) reporting a fall requiring medical attention also reported a fall during their last interview at Wave 5.

### Emergency department attendance with falls

Over 6% (proportion 0.06 (95% CI 0.05 to 0.07)) of participants reported attending the ED with a fall in the last 12 months, including 4% of males (proportion 0.04 (95% CI 0.03 to 0.6)) and 8% of females (proportion 0.08 (95% CI 0.06 to 0.10)).

Figure 1A further demonstrates the breakdown of ED attendance due to falls by age group, with 13% (proportion 0.13 (95% CI 0.09 to 0.18)) of participants aged ≥85 years reporting ED attendance due to a fall in the last year.

Over half (proportion 0.53 (95% CI 0.43 to 0.62)) of participants reporting an ED attendance after a fall were prescribed at least one FRID, while one-fifth (proportion 0.20 (95% CI 0.13 to 0.29)) were prescribed at least two FRIDs. See figure 2.

Almost half (proportion 0.48 (95% CI 0.39 to 0.58)) of participants reporting an ED attendance with falls in the last 12 months also reported a fall during their last interview at Wave 5.

### Fracture

Over 3% (proportion 0.03 (95% CI 0.03 to 0.04)) of participants reported sustaining a fracture (hip, wrist or vertebra) within the last 12 months, including 2% of males (proportion 0.02 (95% CI 0.01 to 0.03)) and 4% of females 0.04 (95% CI 0.03 to 0.06). Over 8% of participants aged ≥85 years reported sustaining a fracture in the last 12 months.

Over half of participants (proportion 0.51 (95% CI 0.37 to 0.63)) reporting a recent fracture were prescribed at least one FRID, while 15% (proportion 0.15 (95% CI 0.08 to 0.27)) were prescribed at least two FRIDs. See figure 2.

Almost half (proportion 0.44 (95% CI 0.31 to 0.58)) of participants reporting a fracture also reported a fall at their last interview at Wave 5.

### Fear of falling

While over one-third of participants (proportion 0.38 (95% CI 0.36 to 0.40)) reported some fear of falling, over 8% of participants (proportion 0.08 (95% CI 0.07 to 0.09)) reported that they were ‘very much afraid’ of falling.

Over 15% of participants ≥85 years reported that they were ‘very much afraid’ of falling.

Over 16% of participants (proportion 0.17 (95% CI 0.15 to 0.19)) reported limiting some of their activities due to fear of falling.

Over half (proportion 0.54 (95% CI 0.46 to 0.62)) of participants who reported that they were ‘very much afraid’ of falling were prescribed at least one FRID, while over one quarter (proportion 0.28 (95% CI 0.21 to 0.36)) were prescribed at least two FRIDs. See figure 2.

### Population-based estimates and regional health areas

When extrapolated to population figures from the 2022 census, we estimate that 61 998 people (95% CI 42 923 to 87 703) in Ireland aged ≥70 years had a fall requiring medical attention within the last year (59% females), while 32 207 older people (95% CI 20 326 to 54,631) had an ED attendance due to a fall (70% females) and 17 288 (95% CI 9237 to 34,031) had a fractured hip, wrist or vertebrae (70% females). We further estimate that 41 366 (95% CI 27 863 to 61 033) people in Ireland aged ≥70 years are ‘very much’ afraid of falling (73% females), while 85 140 (95% CI 65 242 to 1 09 687) limit their activities because of a fear of falling (68% females).

The population estimate numbers of falls in the last year by age group are shown in figure 1B.

**Figure 3:**
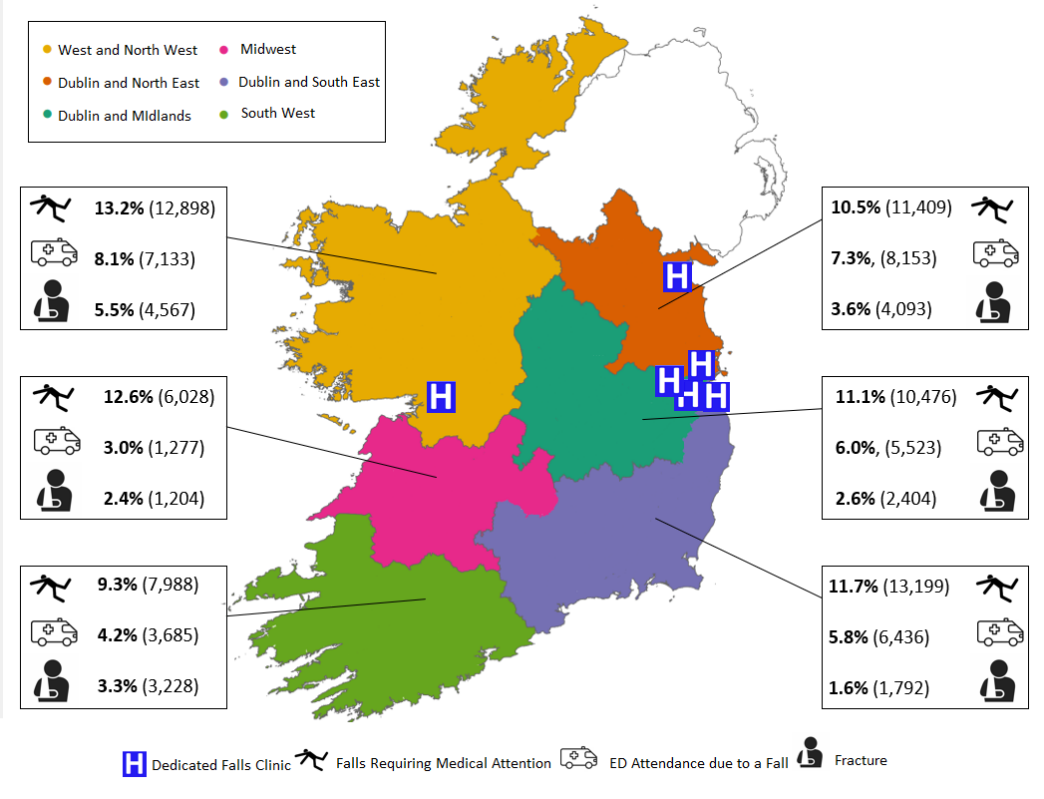
Falls and fractures in people ≥70 years in Ireland in 2022 by proposed regional health areas and dedicated falls clinics. ED, emergency department. Data presented for each regional health area comprise prevalence and estimated numbers of falls requiring medical attention, ED attendance due to a fall and fracture.

Figure 3 further demonstrates falls requiring medical attention, ED attendance due to falls and fractures by RHA and location of dedicated falls clinics. Four of the six RHAs are currently served by dedicated falls clinics. Dedicated falls clinics were in Dublin (St James’s Hospital, Tallaght Hospital, St Mary’s Hospital Phoenix Park/Mater Hospital and St Vincent’s University Hospital), Drogheda (Our Lady of Lourdes Hospital) and Galway (Galway University Hospital). Across the RHAs without a dedicated falls clinic, an estimate of 14 016 older people had a fall requiring medical attention and 4962 presented to the ED with a fall. This equates to 23% of older people with falls requiring medical attention and 15% of those with a falls-related ED attendance being unable to access a dedicated multidisciplinary falls clinic within their RHA.

## Discussion

This study determines the burden of falls among older people in Ireland using population-representative data from TILDA and the 2022 CSO census. We estimate that 1 in 8 people aged ≥70 years sustained a fall requiring medical attention in 2022, and that 1 in 16 attended the ED with a fall during that same timeframe. Currently, this equates to almost 62 000 older people with falls requiring medical attention and almost 35 000 presenting to the ED with falls in 12 months.

Further, 3% of people aged ≥70 years sustained a fracture of their hip, wrist or vertebra within the last year. One in 12 older people reported significant fear of falling, with 1 in 6 or over 80 000 older people reporting that they limited their activities due to fear of falling.

Healthcare activity related to falls is considerable. We found that almost 40% of older people who fell in 2022 required medical attention for a fall and almost 20% of those who fell presented to the ED. A prior study from an ED in North Dublin demonstrated a greater than 60% increase in the numbers of falls-related presentations of older people between 210 and 2014 alone,^32^ while further data show that over 20% of presentations of older people to ED are due to falls.^22^ With the number of older people in Ireland expected to double in the next 25 years,^33^ alongside similar trends globally, it is clear that if the status quo remains, the burden of falls will continue to increase considerably. This work is timely, however, as clinical care in Ireland is currently undergoing a significant transformation and reconfiguration, presenting an opportunity to improve the care of people with falls.

Delivering age-attuned care for patients with falls that meets current and future demand is clearly crucially important, yet there is currently no dedicated clinical programme for falls in Ireland, despite economic analysis demonstrating the net benefits of such a strategy.^30^ Worryingly, only four of the six newly proposed regional health areas were served by a dedicated falls clinic (with four of the six clinics located in Dublin), and therefore over one-fifth of the older people presenting to the ED with falls cannot access a local service dedicated to multidisciplinary falls care. Further, over one quarter of older people with significant fear of falling are also unable to access dedicated falls clinics locally. This is particularly concerning when we show that 1 in 7 older people sometimes limit their activities because of fear of falling. Tailored programmes, particularly physical activity-based interventions, can significantly reduce fear of falling,^34^ as well as co-existing psychological symptoms^35^ however, highlighting the importance of linking older people with fear of falling with appropriate care.

Important, impactful advances have been made in post-fracture care in Ireland in recent years, including the National Hip Fracture Database^36^ and the Irish Fracture Liaison Service Database,^37^ but relatively little progress has been made on falls and fracture prevention over this same timeframe. The morbidity, mortality and healthcare costs associated with an older person sustaining a fracture are considerable.^38 39^ This study demonstrates that half of older people with both falls necessitating an ED visit and falls causing a fracture had a fall within the 2 preceding years.

Additional study findings regarding the use of FRIDs nationally are striking. One in two older people with falls requiring medical attention was prescribed a FRID, while one in five was prescribed at least two FRIDs. Over half of participants with a recent fracture were prescribed at least one FRID, while over one quarter of older people with significant fear of falling were prescribed at least two FRIDs. This is particularly problematic as these cohorts are already at a significantly higher risk of reduced mobility and further falls,^40^ which can be further exacerbated by use of FRIDs via mechanisms such as disturbance of gait and balance, sedation and orthostatic hypotension.^41^

When we consider that 20% of falls can be prevented by a multimodal falls prevention intervention,^42^ and that currently no strategy exists to deliver such an intervention in Ireland, these represent important missed opportunities both at the individual level and for the wider health system. Beyond secondary prevention, our study also demonstrates the need for broader primary falls prevention strategies including falls education and awareness,^43^ promotion of physical activity in later life^44^ and age-friendly design in terms of transport and mobility support and communities and housing.^45^

There are some important limitations to this study that should be noted. Falls were elicited by self-report and therefore there exists a possibility of recall bias; however, it is probably more likely this would lead to under-reporting of falls rather than erroneous reporting,^46^ and falls that require medical attention, that lead to an ED attendance, or a fracture may be less subject to recall bias than all falls. The risk of recall bias in the TILDA study is mitigated by use of informants, if deemed appropriate and with participants’ consent. Additionally, while the study includes participants with a fall requiring medical attention or an ED presentation over a 12-month period, data were not available on recurrent presentations within this same time frame, so the total number of falls/presentations with falls was not available. Data are included on hip, wrist and vertebral fractures but not other fracture types, and there may be information regarding head injuries that was not collected as part of the TILDA study and was therefore unavailable. The context in which patient records are stored within the Irish health system must also be considered, where currently there is no established national electronic health record or unique patient identifier, preventing data linkage with health records and significantly complicating an analysis such as this. This study, therefore, is the first to capture the incidence of falls among older people in both the community and accessing health services. While we use a lower age cut-off of 70 years in this study to ensure alignment with criteria used by many existing services for older people, which define ‘older adult’ populations as beginning at age 70 years, it should be noted that the risk of falls increases from a younger age, with almost one in six people aged 50–64 years in the TILDA study reporting a fall over a 12-month period.^47^

Strengths of the study include the large, population-representative sample of older people followed longitudinally. While this study involves older people from Ireland, findings are applicable to other health systems, including the UK, where provision of falls care is also similarly under-developed.

In conclusion, this study demonstrates the considerable burden of falls among older people in Ireland, with 1 in 8 requiring medical attention for a fall and 1 in 16 attending the ED due to a fall in a 12-month period. Currently, there is no national programme or prevention strategy for falls, and over one-fifth of older people who fall cannot access a dedicated falls clinic locally. While this is particularly concerning given the projected demographic trends, the current reconfiguration of Ireland’s health service represents an important opportunity to improve the care of older people with falls. Strategies to enhance falls prevention are required, both at a population level and in clinical care to prevent the significant morbidity, mortality and healthcare costs associated with falls and fractures.

## Supplementary material

10.1136/bmjopen-2025-107647 online supplemental file 1

## Data Availability

Data are available in a public, open access repository. Data are available upon reasonable request.
