# Supplementary material for: Determining the burden of falls amongst community-dwelling older people in Ireland to inform falls care delivery: secondary data analysis from the Irish longitudinal study on ageing – the defined study
Source: BMJ Open. 2026 Jan 30;16(1):e107647. doi: 10.1136/bmjopen-2025-107647 (PMC12863362; doi:10.1136/bmjopen-2025-107647)
Supplement: online supplemental file 1 [file bmjopen-16-1-s001.docx]

**Supplementary Table 1: Central Statistics Office 2022 Census Data on Older Population in Ireland by Sex and Regional Health Area**

|  | **Age group** | **Total** | **RHA A** | **RHA B** | **RHA C** | **RHA D** | **RHA E** | **RHA F** |
| --- | --- | --- | --- | --- | --- | --- | --- | --- |
| **Male** | *70-74 years* | 99281 | 19120 | 18691 | 19614 | 15490 | 9009 | 17357 |
|  | *75-79 years* | 73726 | 14433 | 13069 | 15253 | 11478 | 6603 | 12890 |
|  | *80-84 years* | 43588 | 8708 | 7128 | 9499 | 6659 | 3813 | 7781 |
|  | *≥85 years* | 31607 | 6228 | 5238 | 6949 | 4803 | 2731 | 5658 |
| **Female** | *70-74 years* | 103603 | 20725 | 19786 | 20835 | 15714 | 9194 | 17349 |
|  | *75-79 years* | 80534 | 16439 | 14377 | 16950 | 12426 | 7220 | 13122 |
|  | *80-84 years* | 52998 | 10963 | 8874 | 11607 | 8218 | 4549 | 8787 |
|  | *≥85 years* | 52834 | 11006 | 8720 | 11712 | 7991 | 4307 | 9098 |
