# Supplementary material for: Determining the burden of falls amongst community-dwelling older people in Ireland to inform falls care delivery: secondary data analysis from the Irish longitudinal study on ageing – the defined study
Source: BMJ Open. 2026 Jan 30;16(1):e107647. doi: 10.1136/bmjopen-2025-107647 (PMC12863362; doi:10.1136/bmjopen-2025-107647)
Supplement: Supplementary file 2 [file bmjopen-2025-107647.reviewer_comments.pdf]

PEER REVIEW HISTORY

BMJ Open publishes all reviews undertaken for accepted manuscripts. Reviewers are asked to complete a checklist review form and are provided with free text boxes to elaborate on their assessment. These free text comments are reproduced below.

ARTICLE DETAILS

Title (Provisional)

The DEFINED Study: Determining the burden of Falls amongst community-dwelling older people in Ireland to Inform Falls Care Delivery. Secondary Data analysis from the Irish Longitudinal Study on Ageing.

Authors

Briggs, Robert; Ward, Mark; Scarlett, Siobhan; van der Velde, Nathalie; Hernandez, Belinda; Romero-Ortuno, Roman; Tysinger, Bryan; May, Peter; Ahern, Emer; Kenny, Rose Anne

---

VERSION 1 - REVIEW

**Reviewer**	**1**
**Name**	**Hill, Anne-Marie**
**Affiliation**	**The University of Western Australia**
**Date**	**25-Sep-2025**
**COI**	**None**

Important topic, as falls prevention requires urgent attention and there is a need to develop a national strategy across multiple countries hence a significant study. Uses a national data base which gives a strong new updated data set for use and further referencing. Relevant outcomes presented -overall a very good study.

Major comments

1. I think more balance needs to be placed into the discussion on management of falls, as multidisciplinary management is required for fallers presenting to ED. Its not clear as the statement says “specialist care” which implies medical care, however comprehensive MDT is the evidence based approach. Additionally, while a clinic is critically important, other national strategies would also be important, such as online websites for older people, local medical practitioner referral for deprescribing, allied health services and public health prevention policy for older people. This should be mentioned in the context of national falls strategies required, in both the introduction and the conclusion, with relevant references.

2. What is the composition and service model of the falls clinics described? Please clarify what services they provide. Is it feasible for all older people who fall to be referred to such a clinic – how does this integrate with Ireland's primary health care system? It might be that falls clinics need community step down services?

Abstract:

Need to add a gender component in the participant setting and would consider re-mentioning in this part of the methods that the setting is Ireland.

The methods need to clarify what prior falls means as wave 5 in the results doesn't hold meaning for international readers. Also the methods don't mention the statistical analysis plan apart from the word "extrapoloated: which is generic – a brief summary of the statistical analysis is standard and is important for readers.

Results: is there a reason the results are presented as a series of stand-alone bullet points? I thought a joined up abstract was required.

Conclusion needs re writing for clarity as the of the second part of the last sentence is not the logical or a contrasting conclusion that flows on from the first part. Two separate sentences would be helpful.

Strengths and Limitations

The strengths need to be written with more clarity, The information on medications needs to mention " falls risk", and isn't it the first population level data for Ireland – need to be specific to Ireland.

Suggest simply give the number of participants and don't say "almost" – with a large sample no need.

The first limitation needs removing and rewriting with another point as reporting in other countries is not a limitation of this study?

"falls are by self report" is not grammatically correct, needs editing.

Visual Abstract – overall the design is suitable for the study but the layout needs clarifying if the reader is to grasp the significance.

The use of Wave 5 is confusing and doesn't allow the results to stand alone – would strongly suggest you remove it. It's not clear where the percentage of those without access to a falls clinic is from? is it those who have the event? Needs clarifying. Also the legend is helpful but too small and the insert icon is small – its hard to properly link the icon to the legend. Icon should be larger and proportion of figure pyramid needs reconfiguring.

Introduction

Line 20 – "this requires…" needs rewording as not clear what is being referred to.

Lines 25 to 30: although reasonable to introduce FRIDS since topic of data collection this background does not mention the myriad of other strategies i.e. strength and balance which presumably you think falls clinics should address. Further background is required with reference to relevant systematic reviews and guidelines to accurately reflect the evidence.

Line 33 "specialist care…" needs to be explained as are you seem to imply medical care? Assume you would mean multidisciplinary team? As falls prevention requires a multidisciplinary approach assume you would advocate for clinics that provide this type of service

Methods

How do you deal with participants who had multiple falls or ED presentations? Needs to be clarified in methods and/ or limitations that the survey doesn't allow for identifying people with multiple falls or ED presentations. If read as described, the results imply there could be undercounting as other studies demonstrate that up to 10/15% of older people will present more than once to ED in 12 months with a fall – is this so in Ireland?

Page 9 line 19 – why those fractures? Rather than other fractures and how would participants know if they were correctly classifying their fracture? Also if self-report why combine into one data point – is this a single question? Clarify for readers not familiar with the TILDA study.

Where do the medication lists come from – participants or their doctors?

Methods also need to explain what access to a falls clinic means, to explain the results. Is the health care system in Ireland meant to provide access to a falls clinic for all older people who attend an ED?

Discussion

Paras 1 and 2 vary with verb tense and need to be adjusted for clarity for the reader.

Line 22 – there is established evidence and guideline description regarding underreporting of falls due to recall bias and this should be referenced here against these statements. It is a known limitation that should be highlighted.

Line 50. need to reference evidence for addressing fear of falling here and describe why it is problematic and how it can be mitigated with treatment, as this is the primary reason why lack of referral is a problem.

Line 13 page 16 rather than leave the reader to try to interpret the significance you need to describe why it is important the people with FoF are prescribed FRIDS – e.g. present evidence about FoF and the risk of falls and injury etc

Conclusion line 49 on – as with the abstract, the second statement in this sentence re needing a national strategy is not a logical conclusion to make from the first part the way it is phrased. Need to make the first point re demographics and point out the need for the service – then talk about the opportunity in another separate statement.

Reviewer 2
Name Ambrens, Meghan
Affiliation Neuroscience Research Australia, Falls, Balance and Injury Research Centre
Date 27-Oct-2025
COI None

Thanks for the opportunity to review this paper. Overall an important piece of work. I have a few comments about decisions you have made and the potential missed opportunity of this paper to focus on prevention.

Introduction

The abstract indicates that there is no current national strategy on falls. Then introduction introduces Slaintecare, however no obvious link is made by the authors to explain this is a government-led health and social care plan. I needed to google this to understand that this is government not a private company.

Further, the focus seems to be on understanding burden of falls to inform better service planning for patients with falls. I think there is a missed opportunity here. Why is there no consideration given to use this data to inform preventive health services?

Study design

Why was the cut off age 70 years when high quality evidence indicates that from 65 years of age “at least one-third of community-dwelling older people” fall each year (Sherrington et al 2019 Cochrane Review)? Other research indicates fall risk at younger ages. This seems like a real limitation that needs to be considered given there are implications related to the provision of services for people who experience a fall.

Analysis

What methods have been used to perform this analysis?

Falls

Why was analysis only conducted on those who reported being very afraid of falling? Research shows that concern about falling increases risk of falls and also limits behaviours such as reducing activities.

You report on fracture (wrist, hip or vertebrae). Why did you not consider head trauma as part of this analysis?

Discussion

I think there is a missed opportunity to discuss the importance of prevention and seeing this “timely” work as an opportunity to inform and shape a preventive-based health policy.

Without preventive measures, our ageing society will continue to fall and overwhelm health services (even those newly developed).

VERSION 1 - AUTHOR RESPONSE

**Reviewer #1**	
I think more balance needs to be placed into the discussion on management of falls, as multidisciplinary management is required for fallers presenting to ED. Its not clear as the statement says "specialist care" which implies medical care, however comprehensive MDT is the evidence based approach.<br><br>Additionally, while a clinic is critically important, other national strategies would also be important, such as online websites for older people, local medical practitioner referral for deprescribing, allied health services and public health prevention policy for older people. This should be mentioned in the context of national falls strategies required, in both the introduction and the conclusion, with relevant references.	Thank you. We agree that falls management requires a comprehensive, multidisciplinary approach, and we appreciate you highlighting that our current wording may unintentionally imply a more medical-only focus. We have changed references of 'specialist care' to multidisciplinary care throughout the manuscript.<br><br>Thank you. We have acknowledged the importance of other strategies in both the introduction and discussion of the revised manuscript. This includes important components of multifactorial falls prevention measures where we have added: 'strategies including strength and balance exercise, 14 home hazard reduction, 15 treatment of vision problems 15 and management of cardiovascular cases of falls,16 have been shown to significantly reduce falls risk' and public health measures: 'Beyond secondary prevention, our study also demonstrates the need for broader primary falls prevention strategies including falls education and awareness, , promotion of physical activity in later life and age-friendly design in terms of transport and mobility support and communities and housing.'
What is the composition and service model of the falls clinics described? Please clarify what services they provide. Is it feasible for all older people who fall to be referred to such a clinic – how does this integrate with Ireland's primary health care system? It might be that falls clinics need community step down services?	Thank you. We have now clarified what dedicated falls clinics are: 'dedicated clinics that provide multifactorial risk assessment for older people with falls via a multidisciplinary team' and noted that 'typically patients can be referred from the community or through secondary/tertiary care. Currently, there is no pathway in place via Ireland's primary care system for older people with falls to have multifactorial falls risk assessment.'
Abstract: Need to add a gender component in the participant setting and would consider re-mentioning in this part of the methods that the setting is Ireland.	Gender breakdown added and now noted that participants Ireland-based.
Abstract: The methods need to clarify what prior falls means as wave 5 in the results doesn't hold meaning for international readers.<br><br>Also the methods don't mention the statistical analysis plan apart from the word "extrapoloated:	Thank you. This has been amended to: '…over half had also reported a fall when assessed at the prior wave of the study (i.e., 2 years ago)'.<br><br>Thank you. This has been amended to: 'Using Central Statistics Office Census 2022, the population of older

which is generic – a brief summary of the statistical analysis is standard and is important for readers.	people in Ireland were multiplied by the proportion of TILDA participants with each outcome of interest to yield population-level estimates.'
Abstract: is there a reason the results are presented as a series of stand-alone bullet points? I thought a joined up abstract was required.	Abstract now joined up.
Abstract: Conclusion needs re writing for clarity as the of the second part of the last sentence is not the logical or a contrasting conclusion that flows on from the first part. Two separate sentences would be helpful.	Thank you. This has been rewritten to: 'Currently, there is no national falls strategy in Ireland. These findings, alongside our ageing population, underscore the need for strengthened falls-prevention strategies, to reduce avoidable morbidity and healthcare utilisation'.
Strengths and Limitations<br>The strengths need to be written with more clarity, The information on medications needs to mention " falls risk", and isn't it the first population level data for Ireland – need to be specific to Ireland.<br>Suggest simply give the number of participants and don't say "almost" – with a large sample no need.<br><br>The first limitation needs removing and rewriting with another point as reporting in other countries is not a limitation of this study?<br>"falls are by self report" is not grammatically correct, needs editing.	Thank you, this section has been fully rewritten as follows:<br>'Strengths<br>•Detailed, population-representative analysis of falls in 2,299 community-dwelling older people with longitudinal follow-up.<br>•Captures proportion of falls requiring medical attention and emergency department attendance at a population-level amongst older people in Ireland, using census data to estimate true burden of falls.<br>•Detailed information on use of falls risk-increasing drugs (FRIDs), cross-checked with individual medication lists.<br><br>Limitations<br>•Data limited to Irish population which may impact on generalizability of findings, however, falls care is similarly underdeveloped across the UK and Europe.<br>•Falls are elicited by self-report so may be subject to some recall bias'.
Visual Abstract – overall the design is suitable for the study but the layout needs clarifying if the reader is to grasp the significance.<br><br>The use of Wave 5 is confusing and doesn't allow the results to stand alone – would strongly suggest you remove it. It's not clear where the percentage of those without access to a falls clinic is from? is it those who have the event? Needs clarifying. Also the legend is helpful but too small and the insert icon is small – its hard to properly link the icon to the legend. Icon should be larger and proportion of figure pyramid needs reconfiguring.	Thank you. Reference to Wave 5 has been removed and text amended for clarity. Pyramid figure has been enlarged for clarify and icons have been replaced with text . Legend has been removed. Reference to falls clinics has also been removed.
Introduction<br><br>Line 20 – "this requires…" needs rewording as not clear what is being referred to.<br><br><br><br>Lines 25 to 30: although reasonable to introduce FRIDS since topic of data collection this background	 <br>Line 20 now reads: 'Falls prevention requires careful clinical assessment, with access to multidisciplinary care for those who require it…'<br><br><br>Thank you. Other important strategies that reduce risk of falls are now noted and referenced in background as

does not mention the myriad of other strategies i.e. strength and balance which presumably you think falls clinics should address. Further background is required with reference to relevant systematic reviews and guidelines to accurately reflect the evidence.<br><br>Line 33 "specialist care…" needs to be explained as are you seem to imply medical care? Assume you would mean multidisciplinary team? As falls prevention requires a multidisciplinary approach assume you would advocate for clinics that provide this type of service	follows: 'Multimodal interventions, which may incorporate strategies including strength and balance exercise, 14 home hazard reduction, 15 treatment of vision problems 15 and management of cardiovascular cases of falls,16 have been shown to significantly reduce falls risk'.<br><br>Thank you. While it was not our intention to imply medical care by use of the term 'specialist care' rather the age-attuned evidence-based multidisciplinary care required for older people with falls, we understand this terminology may cause confusion and have therefore removed it from the manuscript, replaced by multidisciplinary care.
Methods<br><br>How do you deal with participants who had multiple falls or ED presentations? Needs to be clarified in methods and/ or limitations that the survey doesn't allow for identifying people with multiple falls or ED presentations. If read as described, the results imply there could be undercounting as other studies demonstrate that up to 10/15% of older people will present more than once to ED in 12 months with a fall – is this so in Ireland?<br><br>Page 9 line 19 – why those fractures?<br><br>Rather than other fractures and how would participants know if they were correctly classifying their fracture?	Thank you. Unfortunately, the nature of TILDA data collection means we did not capture repeat ED presentations / allow for identification of people with multiple presentations. It is likely that in Ireland, older people who present with falls have similar representation rates. We have noted this in limitations with he following: 'Additionally, while the study includes participants with a fall requiring medical attention or an ED presentation over a 12-month period, data was not available on recurrent presentations within this same time frame, so the total number of falls/presentations with falls was not available.' Our study captures the number of older people who present with falls, rather than the total number of falls however, so we do not undercount as all people with falls leading to an ED presentation are captured.<br><br>This is a secondary data analysis of the TILDA study, which asks specifically about wrist, hip and vertebral fractures (the most frequent 'fragility' type fractures) but does not collect data on other specific fracture types or head injuries, so unfortunately this data was not available. This has been added to limitations.<br><br>Self-report of fractures has been demonstrated to be fairly reliable in previous studies, including one that examined 5-year fracture history (Ivers et al, 2022). As this is a 12-month fracture history, we are satisfied that data is sufficiently robust. This is now noted in methodology.

Also if self-report why combine into one data point – is this a single question? Clarify for readers not familiar with the TILDA study.	TILDA interview questions regarding fractures are now detailed in methodology section. Participants were asked separately about different fractur types. As fracture rates were relatively low, this was combined into a single variable afterwards.
Where do the medication lists come from – participants or their doctors?	Medication lists are provided by participants, this is now clarified in methods.
Methods also need to explain what access to a falls clinic means, to explain the results. Is the health care system in Ireland meant to provide access to a falls clinic for all older people who attend an ED?	Thank you. The following has been added to methods to clarify: 'Currently, there is no pathway in place within Ireland's primary care system for older people at risk of falls to have a multifactorial falls risk assessment, and generally patients are referred on to clinics, e.g. general medical clinics, geriatric medicine clinics, dedicated falls clinics, for the assessment. Data on access to dedicated falls clinics, i.e. dedicated clinics that provide multifactorial risk assessment for older people with falls via a multidisciplinary team, available at each model 3 and model 4 hospital in Ireland were collected.29 Data on clinics is publicly available and listed on the respective hospital referral forms/listings and was confirmed locally' and 'While all older people who fall may not require a multifactorial falls risk assessment, world fall guidelines recommend that any older person who presents to healthcare with a fall causing an injury, ≥2 falls in the last year, frailty, inability to get up from the floor after a fall and/or suspected syncope should have a multifactorial risk assessment.30
Discussion<br>Paras 1 and 2 vary with verb tense and need to be adjusted for clarity for the reader.	Thank you. This has been amended.
Line 22 – there is established evidence and guideline description regarding underreporting of falls due to recall bias and this should be referenced here against these statements. It is a known limitation that should be highlighted.	Thank you. This is highlighted in limitations with appropriate referencing as follows: 'Falls are elicited by self-report and therefore there exists a possibility of recall bias, however it is probably more likely this would lead to under-reporting of falls rather than erroneous reporting and falls that require medical attention, that lead to an ED attendance, or a fracture may be less subject to recall bias than all falls. The risk of recall bias in the TILDA study is mitigated by use of informants, if deemed appropriate and with participants' consent'.

Line 50. need to reference evidence for addressing fear of falling here and describe why it is problematic and how it can be mitigated with treatment, as this is the primary reason why lack of referral is a problem.	Thank you, the following has been added, with appropriate references: ‘Tailored programs, particularly physical activity-based interventions, can significantly reduce fear of falling, as well as co-existing psychological symptoms however, highlighting the importance of linking older people with fear of falling with appropriate care’.
Line 13 page 16 rather than leave the reader to try to interpret the significance you need to describe why it is important the people with FoF are prescribed FRIDS – e.g. present evidence about FoF and the risk of falls and injury etc	Thank you. The following has been added to the discussion, with appropriate references: ‘This is particularly problematic as these cohorts are already at a significantly higher risk of reduced mobility and further falls, which can be further exacerbated by use of FRIDs via mechanisms such as disturbance of gait and balance, sedation and orthostatic hypotension’.
Conclusion line 49 on – as with the abstract, the second statement in this sentence re needing a national strategy is not a logical conclusion to make from the first part the way it is phrased. Need to make the first point re demographics and point out the need for the service – then talk about the opportunity in another separate statement.	Thank you. This has now been divided into separate statements.

**Reviewer #2**	
Introduction<br>The abstract indicates that there is no current national strategy on falls. Then introduction introduces Slaintecare, however no obvious link is made by the authors to explain this is a government-led health and social care plan. I needed to google this to understand that this is government not a private company.	Thank you. The following is added to the introduction with appropriate referencing: ‘Slaintecare is a government-led ten-year plan currently underway to transform publicly-funded health and social care services in Ireland’.
Further, the focus seems to be on understanding burden of falls to inform better service planning for patients with falls. I think there is a missed opportunity here. Why is there no consideration given to use this data to inform preventive health services?	Thank you for this important point. A key component of the falls care we discuss is secondary prevention of further falls and we have now made this more explicit throughout the manuscript, including the conclusion. We have also highlighted the importance of public health measures for falls prevention in the discussion by adding the following, with appropriate references: ‘Beyond secondary prevention, our study also demonstrates the need for broader primary falls prevention strategies including falls education and awareness, promotion of physical activity in later life and age-friendly design in terms of transport and mobility support and communities and housing.’
Study design<br>Why was the cut off age 70 years when high quality evidence indicates that from 65 years of age “at least one-third of community-dwelling older people” fall	Thank you. We agree that fall risk is already substantial from age 65, and that individuals younger than 70 may also experience clinically meaningful falls. Our decision to use 70 years as the lower age cut-off was based on alignment

each year (Sherrington et al 2019 Cochrane Review)? Other research indicates fall risk at younger ages. This seems like a real limitation that needs to be considered given there are implications related to the provision of services for people who experience a fall.	with existing services for older people, which define "older adult" populations as beginning at age 70 to ensure policy and service relevance of the study.<br><br>We have therefore added the following to limitations: 'While we use a lower age cut-off of 70 years in this study to ensure alignment with criteria used by many existing services for older people, which define "older adult" populations as beginning at age 70 years, it should be noted that the risk of falls increases from a younger age, with almost 1 in 6 people aged 50-64 years in the TILDA study reporting a fall over a 12-month period.'
Analysis<br>What methods have been used to perform this analysis?	Thank you. Statistical analysis is outlined in methods. While analysis is mostly descriptive, key statistical methods include appropriate weighting of the sample to ensure population-representativeness and extrapolation to census data to derive population-level estimates, both of which are detailed in this section.
Falls<br>Why was analysis only conducted on those who reported being very afraid of falling? Research shows that concern about falling increases risk of falls and also limits behaviours such as reducing activities.	Almost one third of the TILDA cohort noted some degree of fear of falling and we therefore limited analysis to those with more severe symptoms.<br>We did include all participants who noted that they restricted activities/limited behaviours due to fear of falling however.
You report on fracture (wrist, hip or vertebrae). Why did you not consider head trauma as part of this analysis?	Unfortunately, as this is a secondary data analysis of the TILDA study, data on head injuries/head trauma was not available. We plan to include this in future waves of data collection. This has been acknowledged in limitations.
Discussion<br>I think there is a missed opportunity to discuss the importance of prevention and seeing this "timely" work as an opportunity to inform and shape a preventive-based health policy. Without preventive measures, our ageing society will continue to fall and overwhelm health services (even those newly developed).	Thank you, this is an important message that was under-emphasised in our original manuscript. In this revised version, as outlined above, we have more clearly discussed both primary and secondary prevention of falls including healthcare interventions and public-health measures, as well as noting the importance of prevention in the conclusion of the study.
